# Effect of continuous and intermittent blood flow restriction deep-squat training on thigh muscle activation and fatigue levels in male handball players

**DOI:** 10.1038/s41598-023-44523-7

**Published:** 2023-11-06

**Authors:** Yan Wang, Zhiyuan Li, Che Tongtong, Wenjuan Zhang, Xiaoxiao Li

**Affiliations:** 1 Department of Physical Education Teaching and Research, Fuzhou University, Fuzhou, 350108 Fujian China; 2 Department of Public Physical and Art Education, Zhejiang University, Hangzhou, 310058 Zhejiang China; 3 School of Physical Education, Qingdao University, Qingdao, 266071 China; 4 Department of Military Theory, Fuzhou University, Fuzhou, 350108 Fujian China

**Keywords:** Physiology, Health care

## Abstract

We aimed to investigate acute changes before and after low-intensity continuous and intermittent blood flow restriction (BFR) deep-squat training on thigh muscle activation characteristics and fatigue level under suitable individual arterial occlusion pressure (AOP). Twelve elite male handball players were recruited. Continuous (Program 1) and intermittent (Program 2) BFR deep-squat training was performed with 30% one-repetition maximum load. Program 1 did not include decompression during the intervals, while Program 2 contained decompression during each interval. Electromyography (EMG) was performed before and after two BFR training programs in each period. EMG signals of the quadriceps femoris, posterior femoral muscles, and gluteus maximus, including the root mean square (RMS) and normalized RMS and median frequency (MF) values of each muscle group under maximum voluntary contraction (MVC), before and after training were calculated. The RMS value under MVC (RMS_MVC_) of the rectus femoris (RF), vastus medialis (VM), vastus lateralis (VL), and gluteus maximus (GM) decreased after continuous and intermittent BFR training programs, and those of the biceps femoris (BF) and semitendinosus (SEM) increased; The RMS standard values of the VL, BF, and SEM were significantly increased after continuous and intermittent BFR training (P < 0.05), The RMS value of GM significantly decreased after cuff inflating (P < 0.05). The MF values of RF, VM, VL, and GM decreased significantly after continuous BFR training (P < 0.05). Continuous BFR deep-squat training applied at 50% AOP was more effective than the intermittent BFR training program. Continuous application of BFR induces greater levels of acute fatigue than intermittent BFR that may translate into greater muscular training adaptations over time.

## Introduction

High-load resistance training can effectively promote muscle hypertrophy and increase strength in athletes^1^. Numerous studies have reported that low-load resistance exercise in combination with blood flow restriction (BFR), also known as BFR training, elicits increases in both muscle size and strength, with benefits comparable to traditional high-load resistance training^2–8^. At the same time, BFR training can promote muscle activation to ensure a total power output similar to traditional training^9–11^. In addition, BFR training can enhance neural activation and promote the recruitment of type II motor units^12^. Therefore, the neuromuscular response generated by external pressure stimulation is also one of the main causes of muscle hypertrophy. Shinohara et al. have shown that muscle hypertrophy might be partially driven by neuromuscular responses accompanying BFR training^13^. Nevertheless, whether the neuromuscular component represents a pivotal role in the genesis of muscle adaptations to BFR training is not known.

Previous studies have shown that low intensity compressive resistance training can increase the recruitment and discharge frequency of motor units and activate more fast muscle fibers to participate in muscle activity compared to non-compressive conditions. In addition, the increased electrical activity of muscles can stimulate muscle protein synthesis through the transcription of Ca2^+^phosphatase and calmodulin dependent kinase pathways. Therefore, increasing the intensity of training load can make EMG activity stronger, which is related to an increase in blood lactate concentration and a greater demand for metabolism in muscles. At the same time, as the blood milk concentration increases, the H^+^ concentration also increases, leading to the release of GH and the hypertrophy reaction of fast muscle fibers. It is not yet clear whether neuromuscular factors play a crucial role in muscle adaptation during pressure resistance training. But it is worth affirming that the increase in fast muscle fiber recruitment is a good manifestation of adaptation to muscle hypertrophy caused by low intensity compression training.

In studies on muscle hypertrophy induced by BFR training, different application methods of external pressure resulted in different degrees of fatigue. Moore and Pierce used arbitrary, subjective pressure values to implement a BFR training program^14,15^, while Abe and Yasuda calculated external blood limit pressure based on brachial artery resting systolic pressure and applied it in their experimental design^16,17^. Loenneke et al. pointed out that neither of the above two methods represents an effective strategy for personalizing the pressures of the lower limbs^18^. Patients of different girths experience different degrees of BFR under the same pressure conditions and produce completely different training fatigue responses. Laurentino et al. were the first to use a Doppler probe for determining the pressure required for complete vascular occlusion in the upper thigh at resting conditions^19^. Therefore, the relative pressure calculated using the individual specific percentage of arterial occlusion pressure (AOP) deserves widespread application and promotion.

In studies exploring the instantaneous changes in muscle activation caused by different external pressure stimuli before and after compression resistance training, the exercises were mainly single joint movements such as elbow flexion of the upper limb and isokinetic knee extension of the lower limb. Loenneke et al. found that knee extension with 40–50% AOP to limit blood pressure may change the acute response of the quadriceps femoris and improve the muscle activation level, while higher pressure does not cause these changes^20^. In the field of multi-joint compound movement, Li Zhiyuan et al. concluded that the application of 50% AOP can significantly improve the optimal activation degree of the quadriceps femoris and posterior femoris muscle groups of male handball players at the same time, resulting in the best training effect^21^.

However, the effects of continuous and intermittent BFR training on thigh muscle activation and fatigue during deep squats under the same external adaptive pressure conditions have not been thoroughly investigated. Therefore, this study aimed to investigate the characteristics of the instantaneous changes in muscle group activation and fatigue of the lower limbs of male handball players before and after squat training with two modes of continuous and intermittent BFR training under 50% AOP. This study provides a theoretical basis and reference for the scientific selection and rational utilization of the application mode in BFR training. In this study, we hypothesized that the activation of the anterior and posterior thigh muscle groups increased significantly during both continuous and intermittent BFR training programs.

## Materials and methods

### Participants

Twelve elite national players of the Beijing male handball team were recruited as participants (age: 21.4 ± 3.2 years, height: 190.0 ± 8.7 cm, weight: 91.1 ± 16.2 kg, training years 12.1 ± 2.4 years). Before the experiment, the purpose, method, and possible risks were explained to the participants, and written informed consent was obtained from all participants. Before the test, the essentials of the tested action were explained to the participants, and they were asked to train as usual 1 week before the beginning of the experiment.

### Experimental instruments and equipment

A fully automatic KAATSU Master 2.0 Package (KAATSU Global, Inc., Japan) and 5-cm-wide pressure band were used to digitally display the inflation pressure value. Other instruments included a Wave Plus Wireless surface electromyography (EMG) tester and surface electrodes (Cometa SRL, Italy), a Panasonic HC-V100 Review (Panasonic Global, Inc., Japan), a Gymaware linear sensor device (Kinetic Performance Technology, Australia), a goniometer, a barbell rod and barbell piece, a set of Smith squat racks, a set of tape measures, a metronome.

### Experimental design

#### Test action

Participants stood on the Smith squat rack, with their gaze directed straight ahead, and feet naturally spaced apart. We performed standard squats, characterized by achieving a knee joint angle of 60°–70°, with the thigh roughly parallel to the ground. Both hip and knee were extended simultaneously during the squat movement. The knee joint angle was measured and monitored in real-time using a goniometer, and the participants were prompted verbally. The participants controlled the timing and rhythm of each movement according to the metronome that was set directly in front of the participants.

### Testing method

The athletes’ thigh circumference was measured and evaluated 48 h before the experiment. Due to the limitations of the study equipment, the final pressure was set to a percentage of arterial occlusion estimated from the thigh circumference, and the relative value of the 50% AOP cut-off blood pressure of each person was determined according to the right thigh circumference (Table 1)^20^. The athletes’ right thigh circumference was 62.9 ± 6.1 cm, the occlusion pressure was uniformly selected as 40 mmHg^22^, and the inflatable pressure was 150–180 mmHg. The inflatable pressure of the 5 athletes was 150 mmHg, and that of the other 7 athletes was 180 mmHg. At the same time, the Gymaware linear sensor was used to measure the squat one-repetition maximum (1RM) for each athlete to determine the individualized load intensity (i.e., load weight) for the pressurized squat exercise.

**Table 1:** Blood flow restriction pressures^20^.

Thigh circumference (cm)	Pressure used (60% AOP) (mmHg)	Pressure used (50% AOP) (mmHg)	Pressure used (40% AOP) (mmHg)
< 51	120	100	80
51–55.9	150	130	100
56–59.9	180	150	120
≥ 60	210	180	140

*AOP* estimated arterial occlusion pressure.

Each participant underwent weight-bearing squat exercises under the two intervention conditions of continuous BFR training (Program 1) and intermittent BFR training (Program 2), with a load intensity of 30% 1RM and a relative applied pressure of 50% AOP. Automatic BFR cuffs were applied to the most proximal portion of the participant’s thigh. The cuff was inflated to 50 mmHg for 30 s and then deflated for 10 s. The cuff was then inflated to 100 mmHg for 30 s and then deflated for 10 s (unless 100 mmHg was the target pressure). The cycle of cuff inflation/deflation was repeated with the cuff pressure increasing in increments of 40 mmHg until the target inflation pressure was reached. Before the pressure cuff was inflated to the target pressure, it was pressurized to 50%, 75%, and 100% of the target pressure, respectively, and then the pressure was removed immediately. Next, “inflation-deflation” cyclic pressure adaptation preparation was performed. The cuff was inflated to the target pressure before the first session and removed after the last session. The total duration of each program did not exceed 10 min.

### Testing procedure and collection of indicator data

#### Testing schedule

**Figure 1:**
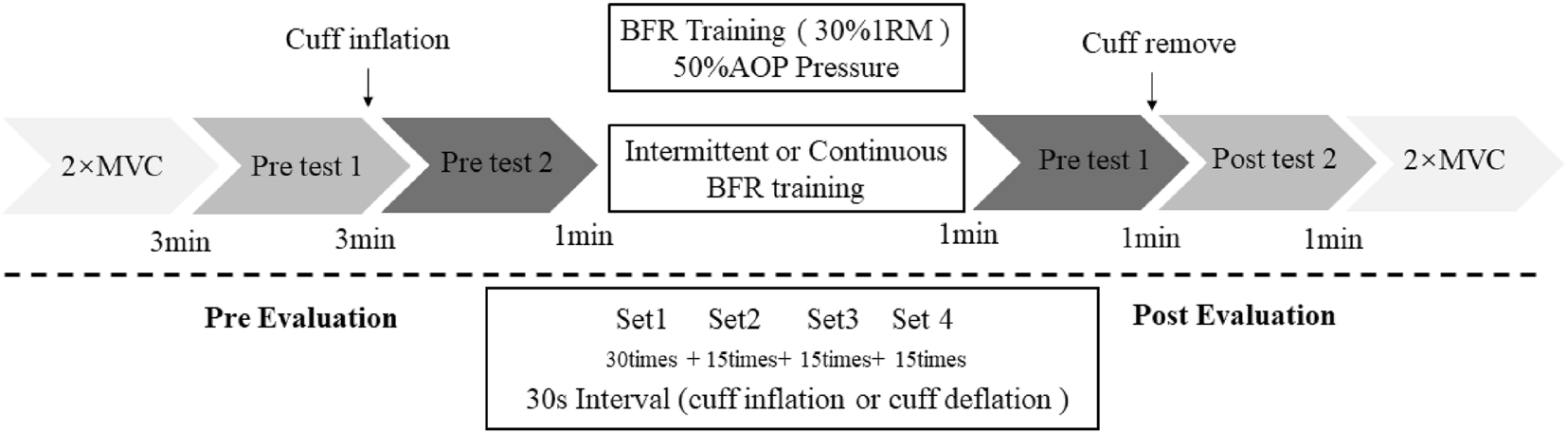
Experimental protocol.

Before the test, participants underwent the following warm-up exercises: (i) 6 min of no-load power cycling (60–70 rpm); and (ii) three sets of 30% 1RM weighted squat exercises (five repetitions per set). After the warm-up, practice and testing were carried out according to the following procedures (see Fig. 1): (i) maximum voluntary contraction (MVC) test of the thigh muscle group; (ii) pre-test 1 (pre-1): three 30% 1RM deep-squat repetitions before cuff inflation; (iii) pre-test 2 (pre-2): three 30% 1RM deep-squat repetitions after cuff inflation; (iv) intermittent and continuous BFR training with 30% 1RM. In both intermittent and continuous BFR training, there were four sets of deep squats with 30, 15, 15, and 15 repetitions intermittent at 30 s. Two types of rest were implemented: keeping pressure and decompression. The interval between the two programs was set to 48 h; (v) post-test 1 (post-1): after implementing the BFT training program, 30% 1RM squat exercises were performed three times; (vi) post-test 2 (post-2): after removing the cuff, three 30% 1RM deep-squat; and (vii) an MVC test of the thigh muscle group.

#### Test indicator data

Gymaware, a linear sensor, was used to test the 1RM of deep squats using the increasing load test method^23^. In the testing procedure, the participants’ maximum strength can be predicted by the linear regression equation: Load = M + B + Z (where M is speed, B is constant, and Z is the standard estimation error) owing to the highly negative correlation between the load and the speed^24^. In the first set, bar speed was more than 1 m/s, and in the last set, the bar speed was less than 0.5 m/s. The number of test sets was approximately 3–5, and the increasing load of each set was 20–30 kg, depending on the weight and strength of the participant.

Thigh circumference was measured with the participants’ feet shoulder-width apart, placing a circumference tape measure at the line below their hips and horizontally measuring their thigh circumference. The left and right thighs were measured three times, and the mean was calculated. The blood pressure limit at each stage was set according to the right thigh circumference.

### Collection and processing of surface EMG data

#### Electrode placement and requirements

The rectus femoris (RF), vastus medialis (VM), vastus lateralis (VL), biceps femoris (BF), semitendinosus (SEM), and gluteus maximus (GM) of the right thigh were selected for testing. The electrodes were placed according to the requirements of the EMG manual^25^; the skin was cleaned before placement, hair was shaved, and the skin was smoothed with sandpaper and then wiped with 75% medical alcohol to remove any oil on the skin’s surface. The surface electrode was placed on the most elevated part of the muscle and fixed along the direction of the muscle fiber to avoid any vibration-induced interference.

### MVC test and root mean square (RMS) value collection

After placing the surface electrodes, the electrode wires were connected to test the thigh muscles’ MVC, and the corresponding muscle surface EMG signals were recorded. Specific testing methods are outlined in the EMG manual^25^. We collected and recorded surface EMG values, which were used as RMS values under the MVC (RMS_MVC_) after processing.

### Surface EMG data collection and processing

Before each squatting test, the camera and Waveplus wireless EMG acquisition system were set up. After the participants began to move, the acquisition system was turned on to collect the EMG data. All EMG signals from 1 to 4 sets of exercise were selected according to the synchronous video of the experiment. The original EMG data were cleaned, filtered, and smoothed, and their amplitude standardized using the matching analysis software of the Emgserver instrument, and the selected index was the RMS amplitude. The range of muscle exertion was selected from the original EMG, and the mean RMS was determined. The EMG RMS of each muscle obtained during MVC testing was defined as 100% MVC. The standardized EMG data processing system automatically divided the EMG RMS value obtained in each set by the EMG of maximum voluntary contraction (EMG_MVC_) value for standardized processing.

### Statistical analysis

Two-way analysis of variance (ANOVA; operation period × intermittent method) and the Mauchly sphericity test were used to evaluate the lower limb muscle groups’ RMS and median frequency (MF) values during the weight-bearing squat in the pre-1, pre-2, post-1, and post-2 periods. If the test P-value was > 0.05, the sphericity test was not satisfied, and the test result of the one-way ANOVA prevailed. If the test P-value was < 0.05, the sphericity test could be satisfied, and two-way ANOVA test results were used. Using the Bonferroni method, multiple comparisons were made between the four operation periods before and after BFR training to test for significant between-condition differences. The RMS_MVC_ was evaluated before and after implementing the two training programs of continuous BFR interval and deflation interval by repeated measurement two-factor analysis (time × interval method). All statistical calculations were computed using the SPSS22.0 statistical software package (SPSS Inc., Chicago, IL) and a significance level of p < 0.05 was used.

### Ethics approval and consent to participate

The study was approved by Fuzhou University Human Research Ethics Committee (LLWYH20200269) and all aspects of the study were conducted in agreement with the Declaration of Helsinki. All of the participants were fully informed about the purpose and experimental procedures of the study. Written, informed consent was obtained prior to the study from each participant. The participants were informed that all data collected would be processed anonymously.

## Results and discussion

### Results

#### Variation characteristics of maximum activation of thigh muscle groups before and after deep-squat training with BFR

As shown in Table 2, the main effects of time on the RF, BF, SEM, and GM RMS_MVC_ values were significantly different (P < 0.05). The time and interval mode interaction were significantly different in the RF, BF, SEM, and GM RMS_MVC_ values (P < 0.05). The RF, VM, VL and GM RMS_MVC_ values decreased after removing the cuff at the end of deep-squat training with BFR. The RF and GM RMS_MVC_ values changed significantly (P < 0.05), while the BF and SEM RMS_MVC_ values increased. There were significant changes in intermittent BFR training (P < 0.05).

**Table 2:** Change in RMS_MVC_ value of thigh muscle group before and after deep-squat training with BFR.

Muscle	Application mode	Pre-test RMS_MVC_	Post-test RMS_MVC_	Relative change (%)	P-value (interaction effect)	P-value (time main effect)
RF	Con	616.32 ± 146.35	548.39 ± 102.37**↓	− 12.39	0.035	0.014
	Int	599.53 ± 107.69	574.37 ± 156.59	− 4.38		
VM	Con	480.94 ± 207.22	444.15 ± 189.95	− 7.65	0.078	0.124
	Int	502.31 ± 147.38	486.39 ± 102.37	− 3.32		
VL	Con	395.68 ± 117.20	369.57 ± 117.56	− 6.60	0.096	0.135
	Int	403.14 ± 123.58	397.23 ± 177.58	− 1.47		
BF	Con	534.05 ± 153.01	591.64 ± 125.03	10.78	0.037	0.039
	Int	514.23 ± 211.58	621.24 ± 255.32**↑	20.81		
SEM	Con	440.58 ± 159.49	449.63 ± 85.21	2.05	0.026	0.034
	Int	476.59 ± 174.53	543.19 ± 97.45**↑	13.97		
GM	Con	422.39 ± 174.41	360.77 ± 213.77**↓	− 5.45	0.047	0.036
	Int	436.15 ± 144.51	412.36 ± 101.25	− 14.59		

Value = mean ± standard deviation.

*BFR* blood flow restriction, *RMS*_*MVC*_ root mean square value under maximal voluntary contraction condition, *RF* rectus femoris, *VM* vastus medialis, *VL* vastus lateralis, *BF* biceps femoris, *SEM* semitendinosus, *GM* gluteus maximus, *Con.* continuous BFR training, *Int.* intermittent BFR training. The same as following tables.

Significant differences were set at p < 0.05(*) for RMS_MVC_ value between pre-test and post-test.

As shown in Table 3, there was a significant difference (P < 0.05) in the main effect of operation time on the RMS standard values of the VM, VL, BF, SEM, and GM. There were significant differences in the RMS standard values of the VM, BF, SEM, and GM between the two modes (P < 0.05). According to multiple comparison results in Table 3, the standard RMS value of the anterior thigh muscle group increased in the pre-2, post-1, and post-2 periods during continuous and intermittent BFR training compared with that in the pre-1 period. The RMS value of VL showed significant changes in the post-1 period (P < 0.05), and the RMS value of the VM and VL had significant changes in the post-1 period (P < 0.05) during intermittent BFR training. The RMS standard values of the posterior thigh muscle group were increased in the pre-2, post-1, and post-2 periods, and the RMS standard values of the BF and SEM were significantly changed in the post-1 period (P < 0.05). The RMS standard value of the semitendinosus changed significantly compared to that in the pre-2 period (P < 0.05). During continuous and intermittent BFR training, the RMS standard value of the GM decreased significantly in the pre-2 period compared with that in the pre-1 period (P < 0.05), while the RMS standard value of the GM increased significantly in the post-2 period compared with that in the pre-2 period (P < 0.05).

**Table 3:** Change in RMS (%MVC) of thigh muscle group before and after deep-squat training with BFR.

Muscle	Application mode	Period				P-value (interaction effect)	P-value (time main effect)
		Pre-1	Pre-2	Post-1	Post-2		
RF	Con	38.10 ± 9.68	46.32 ± 18.32	44.33 ± 17.30	43.31 ± 7.93	0.855	0.074
	Int	40.10 ± 7.38	44.32 ± 14.35	46.33 ± 7.75	45.31 ± 14.44		
VM	Con	49.62 ± 2.74	50.28 ± 6.52	57.31 ± 7.81	55.68 ± 8.91	0.042	0.031
	Int	52.62 ± 4.66	53.32 ± 12.35	58.31 ± 10.58*	57.82 ± 12.60		
VL	Con	46.25 ± 2.56	50.28 ± 6.52	56.25 ± 2.83*	50.63 ± 5.70	0.769	0.014
	Int	49.31 ± 5.77	52.14 ± 9.09	57.87 ± 11.47*	54.41 ± 15.50		
BF	Con	20.48 ± 7.82	23.52 ± 3.41	26.80 ± 4.51*	24.11 ± 3.50	0.025	0.048
	Int	18.55 ± 9.24	22.52 ± 13.72	28.38 ± 9.63*	26.39 ± 9.87		
SEM	Con	19.09 ± 4.75	20.31 ± 5.57	28.20 ± 4.26*^∆^	24.53 ± 7.11	0.031	0.036
	Int	21.56 ± 8.59	21.93 ± 9.81	25.97 ± 7.37*^∆^	23.37 ± 11.85		
GM	Con	31.04 ± 8.75	27.34 ± 8.74*	29.73 ± 14.56	32.29 ± 14.33^#^	0.027	0.022
	Int	34.88 ± 2.90	29.04 ± 4.70*	28.53 ± 13.29	30.30 ± 4.40^#^		

RMS (%MVC) = standard value of root mean square.

*RF* rectus femoris, *VM* vastus medialis, *VL* vastus lateralis, *BF* biceps femoris, *SEM* semitendinosus, *GM* gluteus maximus, *Con.* continuous BFR training, *Int.* intermittent BFR training.

*Indicates that pre-2, post-1, and post-2 are significantly different from PRE-1; ^∆^indicates a significant difference between post-1 and post-2 compared with pre-2; ^#^indicates that post-2 is significantly different from post-1.

#### Comparative analysis of fatigue of the thigh muscle group before and after the compression squat exercise

As shown in Table 4, the main effect of the operation period on MF values of the anterior thigh muscle group and GM were significantly different (P < 0.05). In contrast, the interaction between the operation period and intermittent mode showed a significant difference in MF values of the anterior thigh muscle group and GM (P < 0.05). The multiple comparison results (Table 4) show that, compared with pre-1, the MF values at pre-2, post-1, and post-2 show a trend of an initial increase followed by a decrease in the two schedules of continuous BFR training and intermittent BFR training. The MF values of the RF, VM, and VL decreased significantly during the post-1 period (P < 0.05). However, in the post-2 period, the MF values of the RF and VM returned to levels of the pre-1 period. There were no significant changes in the MF values of BF and SEM in the pre-2, post-1, and post-2 periods (P > 0.05). After continuous BFR training, the MF value decreased significantly in post-1 compared to pre-1 (P < 0.05).

**Table 4:** Change in MF value of thigh muscle group before and after deep-squat training with BFR.

Muscle	Application mode	Period				P-value (interaction effect)	P-value (time main effect)
		Pre test-1	Pre test-2	Post test-1	Post test-2		
RF	Con	66.21 ± 11.47	65.17 ± 11.07	57.13 ± 6.43*^∆^	65.00 ± 7.94^#^	0.011	0.028
	Int	65.15 ± 11.22	64.13 ± 11.40	60.10 ± 8.28	63.90 ± 7.50		
VM	Con	58.22 ± 5.96	59.50 ± 5.70	52.11 ± 5.22*^∆^	58.83 ± 5.80^#^	0.037	0.040
	Int	56.74 ± 6.70	57.47 ± 5.66	52.07 ± 6.27	55.61 ± 6.07		
VL	Con	60.92 ± 6.45	61.17 ± 6.46	56.03 ± 5.07*^∆^	59.58 ± 7.23	0.035	0.047
	Int	58.88 ± 4.62	59.04 ± 6.45	57.07 ± 4.53	59.58 ± 7.23		
BF	Con	62.97 ± 10.13	60.75 ± 9.80	64.68 ± 12.24	63.36 ± 9.59	0.748	0.102
	Int	64.92 ± 8.18	62.25 ± 7.17	66.68 ± 11.44	63.36 ± 9.59		
SEM	Con	65.67 ± 13.22	65.31 ± 9.75	69.54 ± 8.58	67.30 ± 11.11	0.804	0.097
	Int	67.73 ± 13.02	66.20 ± 9.25	69.51 ± 9.60	67.17 ± 7.89		
GM	Con	42.62 ± 4.65	41.16 ± 3.71	37.31 ± 3.98*	40.99 ± 4.69	0.043	0.019
	Int	44.52 ± 3.96	43.12 ± 3.28	45.27 ± 5.62	44.97 ± 7.25		

*MF* median frequency, *BFR* blood flow restriction, *RF* rectus femoris, *VM* vastus medialis, *VL* vastus lateralis, *BF* biceps femoris, *SEM* semitendinosus, *GM* gluteus maximus.

*Indicates that pre-2, post-1, and post-2 are significantly different from PRE-1; ^∆^indicates a significant difference between post-1 and post-2 compared with pre-2; ^#^indicates that post-2 is significantly different from post-1.

#### Characteristics of changes in activation of lower limb muscle groups by deep-squat training with BFR

**Figure 2:**
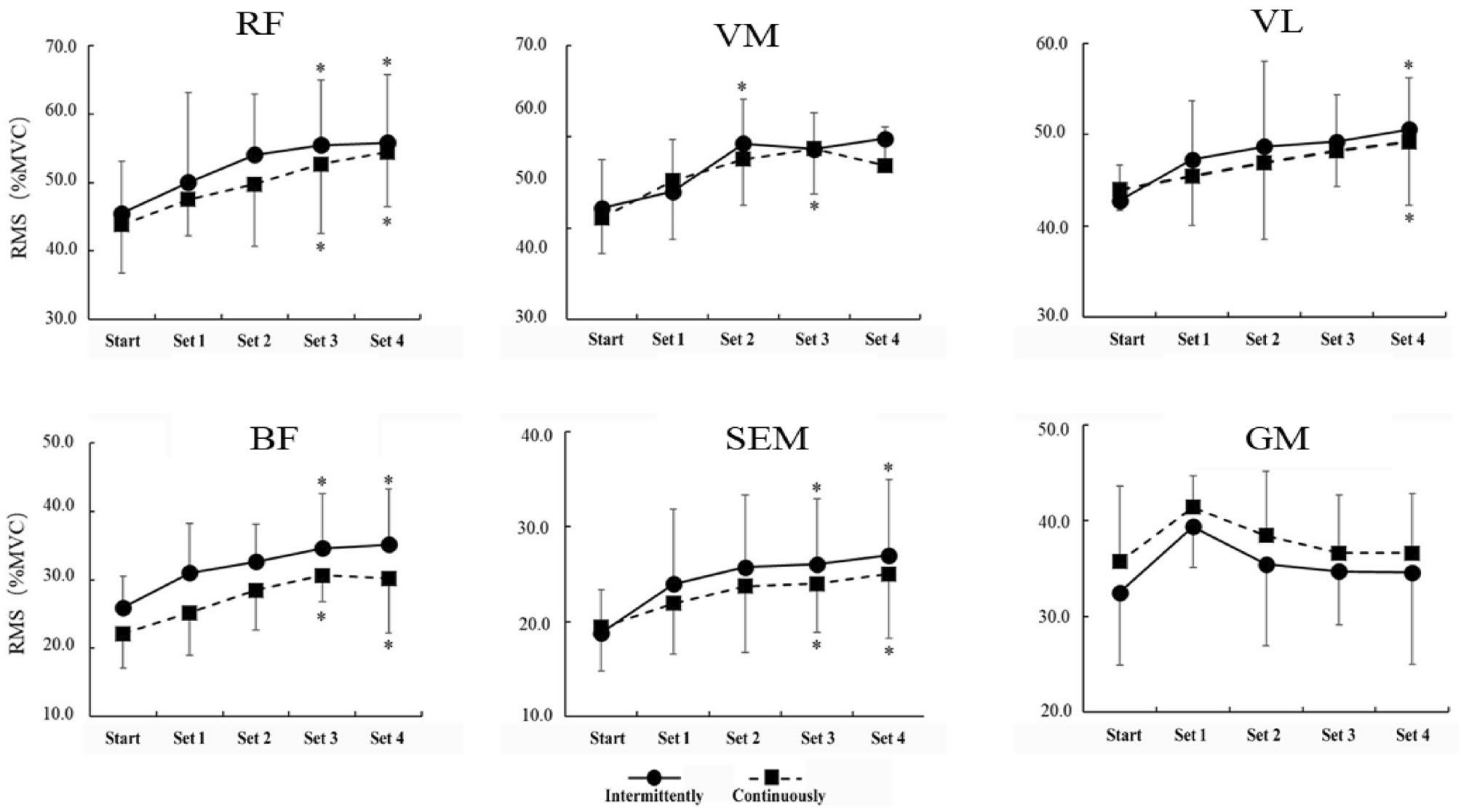
The change in the RMS (%MVC) of the thigh muscle group during the KAASTU deep-squat exercise. *Indicates significant differences between sets 1, 2, 3, and 4 compared with the beginning period. *RF* rectus femoris, *VM* vastus medialis, *VL* vastus lateralis, *BF* biceps femoris, *SEM* semitendinosus, *GM* gluteus maximus.

As shown in Fig. 2, under the application modes of continuous and intermittent, in sets 1–4, the activation of the RF, VM, VL, BF, SEM, and GM increased compared with the initial stage in pre-2. Furthermore, the activation of the RF, BF, and SEM increased significantly in the third and fourth sets (P < 0.05). The activation of VL increased significantly only in the fourth set (P < 0.05), while VM activation increased significantly in the fourth set of continuous BFR training and the third set of intermittent BFR training (P < 0.05). The activity of the GM did not change significantly between the first and fourth sets in either continuous BFR training or intermittent BFR training (P > 0.05).

### Consultation

#### Change characteristics of the thigh muscle groups’ maximum activation before and after compression squat exercises

The results in Table 2 show that the RMS_MVC_ values of the RF, VM, and VL decreased after cuff deflation. The RMS_MVC_ values of the RF changed significantly after continuous BFR training (P < 0.05), while those of the VM and VL showed no significant change (P > 0.05). Most previous studies showed that after the implementation of BFR training, the maximum activation of active muscles restricted by blood flow had a downward trend. Loenneke et al.^18^ performed BFR and non-BFR unilateral knee extension exercises with the quadriceps as the main active muscle group in 16 healthy adult males and found that the MVC value of the quadriceps muscle decreased after leg extension exercises with BFR and non-BFR. Umbel et al.^26^ found that the MVC value of concentric contraction decreased by 9.8%, and the MVC value of concentric contraction decreased by 3.4% after 24 h of unilateral knee extension exercises with BFR and returned to normal after 96 h, which may be related to delayed onset muscle soreness. Fatela et al.^27^ performed knee extension exercises with different BFRs in 14 adult males and found that the RMS_MVC_ values of the VM and VL decreased significantly after exercise under 60% BFR and 80% BFR. This study found that compared with knee extension exercise, the squat exercise with BFR can lead to reduction of quadriceps activation. Additionally, compared to the intermittent BFR training, continuous squat training with BFR led to more reduction, which may be related to acidosis caused by excessive accumulation of metabolite in active muscle cells.

In addition, the results in Table 2 also show that the RMS_MVC_ values of the BF and SEM increased after squat training with BFR, and there was a significant change after the intermittent training. However, the RMS_MVC_ value of the GM decreased and significantly changed after continuous BFR training. The results showed that the RMS_MVC_ of the posterior thigh muscle showed an increasing trend after squat training with BFR. Yasuda et al.^28^ also found that the activation contribution rate of the triceps brachii as an antagonistic muscle increased from 40 to 60% after 30%-1RM multi-joint bench press exercise with BFR, suggesting that BFR training can improve the maximum independent activation rate of antagonistic muscles, thus effectively developing the strength of antagonistic muscle groups compared with multi-joint exercise without BFR. The results also showed that RMS_MVC_ values of the BF and SEM increased significantly after BFR training. This indicated that BFR training can provide an optimal acidic environment to activate type II motor units in the posterior thigh muscle group.

#### Changes in the activation degree of thigh muscle groups before and after squat training with BFR

Many studies have confirmed that the RMS value of active muscles increases during low-intensity BFR training. After BFR training, the RMS values of the anterior and posterior thigh muscle groups were higher than those before BFR training. During continuous BFR training, the RMS values of VL, BF, and SEM were significantly increased. After the cuff was removed, the RMS values of the anterior and posterior thigh muscle groups decreased compared with the RMS values of the period before the inflation at the end of the implementation of the BFR program, but were higher than the RMS values after inflation.

The above results show that after the implementation of the BFR training program, activation of the thigh’s anterior and posterior muscle groups increased. This is mainly caused by the acidic environment resulting from the accumulation of metabolites in the muscle, which can activate more type II muscle fibers, thus increasing the RMS value of the EMG signal on the surface^29^. Fatela et al.^27^ also found that the activation level of the RF and VM changed during knee extension exercises with BFR. Acute BFR stimulation increased the RMS values of the RF and VM; nevertheless, the RF and VM responded differently to different BFR stimulations. The activation level of the VM was significantly increased in 60% and 80% BFR before BFR training (post-1) compared to that at pre-2. The activity of the RF increased significantly only under 80% RFR, suggesting that higher blood pressure restriction can induce a reflex increase in VM and RF activation. The results also showed that the VL, BF, and SEM had similar characteristics of change before and after inflation with continuous and intermittent training under continuous BFR, and that in the VL, BF, and SEM, intermittent training significantly increased VM activation. In addition, the results in Table 3 show that the activation of the GM was significantly reduced after cuff removal. It was comparable to that after cuff removal at the end of the treatment and did not return to pre-inflation levels after intermittent training. Sun et al.^30^ concluded that low-intensity BFR training and hard pull exercises can increase the activation degree of the distal muscles with BFR and the proximal muscles with no limited synergistic function. This study showed that low-intensity squats with BFR reduced the activation of the GM, suggesting that different BFR training had different effects on the activation of unrestricted proximal coordination muscles.

#### Changes in fatigue degree of thigh muscle groups before and after squat training with BFR

Indicators reflecting muscle fatigue are usually expressed by the MF, and the decrease in the MF value is strongly correlated with the decrease in the swing rate of the muscle bridge^31^. Place et al.^32^ confirmed that the degree of intramuscular acidity and the reduction of Ca^2+^ absorption by the sarcoplasmic reticulum are the main factors affecting the decline in muscle contraction function. The results in Table 4 show that the MF values of the RF, VM, and VL decreased before the end of BFR training in both the continuous and intermittent programs, and there were significant differences after continuous BFR training mode. It is suggested that the primary muscle in the front group of the thigh is the main force-generating muscle group, which leads to the excessive accumulation of metabolites such as lactic acid in the muscle during the weight training in the continuous BFR mode. This results in acidity changes, reduced Ca^2+^ absorption by the sarcoplasmic reticulum, and ultimately, accelerated muscle fatigue.

After a short rest of 1 min after deflation, the MF values of the three muscles in the front thigh group recovered to the pre-inflation level. This indicates that the functional decline of the muscle caused by the two programs at 50% AOP level is temporary, and it can be restored to the level before inflation after a short rest with deflation. In addition, Pierce et al.^15^ pointed out that 60% AOP of continuous knee extension exercise with BFR can lead to a significant reduction in the MF values of VL and RF. Neto et al.^33^ also performed a set of 80% 1RM high-intensity squats with 60% AOP, and the results showed that the MF values of the VM and VL decreased by 18.5% and 18.2%, respectively. Previous studies have shown that BFR training induces fatigue mainly by stimulating protein synthesis of the Akt/mTOR signaling pathway, and the decrease in MF values is sensitive to biochemical changes in type II muscle fibers^34^.

Combined with the above results, it can be concluded that neuromuscular fatigue is affected by the intermittent mode and the external BFR intensity. A higher BFR intensity will cause greater fatigue and slower recovery speed. In addition, Table 4 shows that the MF values of the BF and SEM increased before cuff removal at the end of BFR training in both modes and returned to the level before cuff removal 1 min later. This also suggests that both continuous and intermittent modes of low-load squat exercise with BFR can largely promote the activation of the posterior thigh muscle group, ensure that the neuromuscular component is at a suitable level of excitement, ensure the high synchronization of motor neuron discharge, and then promote the reflex improvement of contraction force. In addition, the two modes of low-load squat exercise with BFR had different effects on muscle fatigue in the unrestricted area. The MF value of GM decreased significantly after continuous BFR training but recovered shortly after deflation, while intermittent BFR training had less effect on the MF value of GM. It does not cause fatigue in the GM.

#### Change characteristics at the activation of lower limb muscle groups in each set of squat training with BFR

As shown in Fig. 2, anterior thigh muscle group activation was higher during all four sets of squats with BFR than before the experiment, suggesting that the RF, VM, and VL were activated to varying degrees during each set of compression. The acidic environment created after the application of BFR stimulation can attract more type II motor units to participate in the activity, so that the discharge frequency of fast muscle motor units gradually increases. The RMS value of the RF in the third and fourth sets was significantly higher than that in the initial set (P < 0.05). The RMS values of the VM were significantly higher in the second set of continuous BFR training and the third set of intermittent BFR training than at the initial stage (P < 0.05), and the RMS value of the VL was significantly higher in the fourth set of both modes of practice than at the initial stage (P < 0.05). Counts et al.^35^ found that the degree of activation of the biceps brachii during an elbow flexion exercise was not affected by BFR. High-BFR and low-BFR had similar adaptive effects on muscle hypertrophy, strength, endurance, and other aspects, but high-BFR could produce higher discomfort. In addition, Dandel et al.^36^ found that adding BFR into intervals of high-intensity elbow flexion training could not promote the activation of the biceps brachii and cause muscle hypertrophy. It has been suggested that BFR during low-load squat training can promote muscle activation and cause a hypertrophic response. The results of this study, similar to previous studies, showed that continuous and intermittent training of the upper and lower extremities with moderate intensity blood pressure limitation could effectively improve the activation degree of the prime muscle and reduce discomfort.

Figure 2 shows that the activation of BF and SEM was significantly higher in the third and fourth sets than at the beginning of the squat training with BFR. However, activation of the GM increased in the first set. Activation in the second, third, and fourth sets gradually declined, indicating that both continuous and intermittent BFR training not only improved the thigh before a dynamic degree of muscle activation, but also improve the state of no pressure and rarely participate in and mobilization of the stocks after the muscle group, such as the degree of antagonist muscle activation. This can improve the thigh muscle group compared to flexion and extension and prevent sports injuries. In addition, Abe et al. used 20% 1RM for squat training, believing that, due to the low intensity and the unrestricted position of the GM, such loads would not cause the hypertrophy of the GM^37^. However, there may be synergies between the thigh and buttocks in squat training. This study showed that the GM was complementary, and its activation increased significantly when the anterior thigh group was relatively low during BFR training. In the second, third, and fourth exercise sets, the activity of the anterior thigh muscles gradually increased, while the activity of the GM gradually decreased. This may be because the GM extends the hip while the quadriceps muscle mainly extends the knee. The two muscles may be complementary in function to some extent as the adjacent active muscles of the important lower limb motor chain and the GM may recruit more motor units to supplement the quadriceps deficit. This demonstrates that 50% AOP during squat training with BFR can induce more significant muscle group activation in the front and back of the legs, while GM stimulation was not significant for hip extension. The authors believe that the main reason for this is that the inflation site mainly plays a major role in lower limb blood flow occlusion but has little effect on the gluteal muscle. At the same time, lightweight squat training may not significantly affect the GM, and further extensive hip extension or increased weight-load to 85% or more may induce greater GM activation.

### Limitations

Due to the limitations of research conditions, considering the daily training of sports teams, the accumulated effects of the athletes' other physical and technical training may have interfered with the subsequent tests to a certain extent and affected the accuracy of the acquired EMG data. In addition, the study did not measure the blood concentrations of lactic acid, creatine phosphate, or other blood metabolites or the participants’ subjective physical effort level. To meet the need for training practice, future studies should combine the methods of electric index blood physiological and biochemical indicators and subjective indicators, which could more comprehensively evaluate the mechanism and effect of BFR training.

### Preprint

A previous version of this manuscript was published as a preprint^38^.

## Conclusions

In 30% 1RM squats with 50% AOP, the acute activation of thigh muscle groups was different between continuous BFR training and intermittent BFR training. The degree of high muscle groups activation induced was greater in continuous BFR training than in intermittent BFR training. The RMS values of all the muscle groups increased significantly after training in the third and fourth sets except the VM, but fatigue also occurred. In contrast, although the intermittent mode of deflation produces fatigue, recovery is faster. Therefore, the use of a continuous BFR training mode is recommended for trained athletes and an intermittent BFR training mode for beginners.

## Data Availability

The datasets used or analyzed during the current study are available from the corresponding author on reasonable request.
